# Clustering metagenomic sequences with interpolated Markov models

**DOI:** 10.1186/1471-2105-11-544

**Published:** 2010-11-02

**Authors:** David R Kelley, Steven L Salzberg

**Affiliations:** 1 Center for Bioinformatics and Computational Biology, Institute for Advanced Computer Studies, College Park, MD 20742, USA; 2 Department of Computer Science, University of Maryland, A.V. Williams Building College Park, MD 20742, USA

## Abstract

**Background:**

Sequencing of environmental DNA (often called metagenomics) has shown tremendous potential to uncover the vast number of unknown microbes that cannot be cultured and sequenced by traditional methods. Because the output from metagenomic sequencing is a large set of reads of unknown origin, clustering reads together that were sequenced from the same species is a crucial analysis step. Many effective approaches to this task rely on sequenced genomes in public databases, but these genomes are a highly biased sample that is not necessarily representative of environments interesting to many metagenomics projects.

**Results:**

We present SCIMM (Sequence Clustering with Interpolated Markov Models), an unsupervised sequence clustering method. SCIMM achieves greater clustering accuracy than previous unsupervised approaches. We examine the limitations of unsupervised learning on complex datasets, and suggest a hybrid of SCIMM and supervised learning method Phymm called PHYSCIMM that performs better when evolutionarily close training genomes are available.

**Conclusions:**

SCIMM and PHYSCIMM are highly accurate methods to cluster metagenomic sequences. SCIMM operates entirely unsupervised, making it ideal for environments containing mostly novel microbes. PHYSCIMM uses supervised learning to improve clustering in environments containing microbial strains from well-characterized genera. SCIMM and PHYSCIMM are available open source from http://www.cbcb.umd.edu/software/scimm.

## Background

Over the last 15 years, DNA sequencing technologies have advanced rapidly, allowing sequencing of over one thousand microbial genomes [1]. Still, this accounts for only a sliver of the fantastic diversity of microbes on the planet [2]. Sequencing of environmental DNA (often called metagenomics) has shown tremendous potential to drive the discovery and understanding of the "unculturable majority" of species -- the vast number of unknown microbes that cannot be cultured in the laboratory [3]. Successful metagenomics projects have sequenced DNA from ocean water sampled from around the world [4], microbial communities in and on humans [5-8], and acid drainage from an abandoned mine [9]. These and many other projects (e.g. [10-12]) promise to uncover the true extent of microbial diversity and give us a better understanding of how these unknown microbes live.

However, progress has been slowed by the difficulty of analysis of metagenomic data. The output from an environmental shotgun sequencing project is a large set of DNA sequence "reads" of unknown origin. Because these reads come from a diverse population of microbial strains, assembly produces a large collection of small contigs (contiguous stretches of unambiguously overlapping reads) [13,14]. Two important goals of metagenomics are to determine what species are in the mixture in what proportions and to assemble substantial portions of individual genomes. A fragmented assembly of short sequences makes attaining these goals difficult. Advances in computational analysis techniques are essential to move the field forward.

To uncover what microbes are in a metagenomic sample, we must determine (1) which sequencing reads came from the same microbial strain, and (2) where those strains fit into the phylogenetic tree of life [15]. Methods to solve these two problems are related. Clustering methods solve the former problem by binning sequences into clusters that represent a single taxonomic class. classification methods aim to solve the latter problem by assigning a specific taxonomic class to every sequence.

In some cases, the presence of marker genes like 16 S rRNA, which is very highly conserved across species but has variable regions, can be used to assign a taxonomic class to sequence fragments [16,17], but this typically pertains to only a very small percentage of the reads. For example, ~0.1% of reads in a typical metagenomics project carry rRNA genes [15]. More general sequence similarity-based methods align reads with BLAST [18] to known genomes deposited in public databases like GenBank [19] and use those alignments to assign a taxonomic classification [20-22]. However, sequence alignment can only classify reads from organisms with a close evolutionary relative that has already been sequenced [23]. In most environments, this will not be the case for many of the reads; e.g. 70% of Sargasso Sea reads had a BLAST hit using "extremely lenient" search parameters, and only 30% aligned for nearly their whole length [4].

Composition-based methods for clustering and classification use properties of the DNA sequence such as oligonucleotide frequencies. These "genome signatures" are influenced by a variety of factors including codon usage, DNA structure, replication and repair processes, and evolutionary pressures [24-26]. They are fairly constant within a genome [27-29] and can be useful for inferring phylogenies [30]. Crucially for the use of genome signatures for clustering and classification, they persist even in conserved [31] or horizontally transferred regions (after a sufficient period of time) [32] and remain diverse between species despite shared environmental pressures and interactions [33]. Composition-based classification methods typically train on the oligonucleotide frequencies of all known genomes, and then classify sequences using supervised machine learning such as kernelized nearest neighbor [34], support vector machines [35], self-organizing maps [36], and naive Bayesian classifiers [37]. Phymm, a recently developed composition-based approach developed in our group [38], trains interpolated Markov models (IMMs) on known genomes in order to classify sequences.

While supervised learning has proven useful in practice, shortcomings exist. Methods trained on the genomes in GenBank make an implicit assumption that those genomes are representative of microbes waiting to be found by metagenomics projects. This assumption is clearly violated by many if not most metagenomic samples. Supervised learning methods that tread carefully with respect to the potential biases caused by this assumption can still be useful analytical tools for many environments. Alternatively, genome signatures can be used for unsupervised clustering by learning the signatures from the set of sequences without the use of known genomes [33,39-42]. Such approaches may be required when publicly available genomes are a poor fit to the data.

As an alternative to oligonucleotide frequencies, Markov chain models have shown great promise for characterizing genomic content [43], and have been implemented for both supervised classification [38] and unsupervised clustering [40] methods. In this paper, we cluster sequences using interpolated Markov models (IMMs), a type of Markov chain model that adapts the model complexity to take advantage of variable amounts of training data. This strategy is well suited to metagenomics clustering problems, where the amount of sequencing performed and the relative abundances of the species in the mix can vary widely. Our clustering framework proceeds similarly to one used to cluster sequences using hidden Markov models where optimization is performed iteratively by a relative of the *k*-means clustering algorithm [44]. We refer to our method as SCIMM (Sequence Clustering with Interpolated Markov Models).

We test Scimm on simulated metagenomic datasets of fragments from mixtures of randomly selected known genomes and demonstrate improvement on the performance of the metagenomic sequence clustering programs CompostBin [39] and LikelyBin [40]. We also assess the limitations of unsupervised learning on complex datasets, and describe how a combination of Scimm and Phymm, which we call PhyScimm, clusters more accurately when useful training data is available.

## Methods

Markov models have proven to be an invaluable tool for sequence analysis [45], including capturing genome signatures [43]. Here we present a clustering algorithm called SCIMM in which we use interpolated Markov models (IMMs) to model clusters of sequences. Clustering of sequences is performed using a variant of the *k*-means algorithm.

### Interpolated Markov models

A fixed-order Markov chain is a model for generating a sequence of outputs (in this case, nucleotides in a DNA molecule) in which the *i*^th ^element in the sequence has a distribution that is conditional on the previous *w *elements. Thus, given a sequence s and a model *m*, we can compute the probability that s was generated by *m *by walking along the sequence and multiplying the conditional probabilities.

(1) $$P(s|m)=\prod_{i=w+1}^{\left|s\right|}P_{m}(s_{i}|s_{i-1}s_{i-2}...s_{i-w})$$

**Figure 1:**
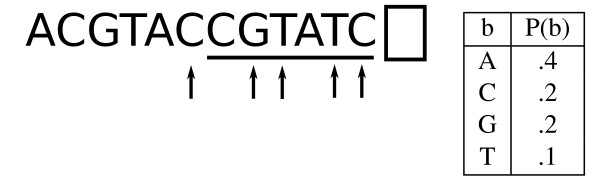
**Markov models**. In a standard *w*^th^-order Markov chain model, the next base *b *in the DNA sequence is assigned a probability that is conditioned on the previous *w *bases (underlined above for *w *= 6). *w *should be chosen so that the data contains a sufficient number of instances of all 4*^w ^*substrings of length *w*. An IMM uses all of the Markov models from order 0 to *w *and computes the probability of the next base by interpolating among them. Our version of the IMM takes this a step further: rather than using the *w *immediately preceding positions, we use the most "informative" positions (shown above with arrows) of the previous *w *according to a recursive mutual information calculation.

Alternatively, IMMs are variable-order Markov chains, a strict generalization of fixed-order Markov chains, and interpolate between multiple models of fixed size via weights (also referred to as "model averaging"). Past work has found that increasing the order of the Markov model (e.g., using a 3^rd^-order model instead of a 2^nd^-order model) leads to more accurate predictions as long as there is sufficient training data. IMMs dynamically adjust the order of the models based on the data, which allows them to make the most of whatever information is available. This is particularly useful for clustering of metagenomic sequences where the amount of sequence from each species may differ widely due to differential abundance of organisms and the amount of sequencing performed on the sample. The variant of IMMs used in our system, introduced in the Glimmer 2.0 gene prediction software [46], is even more general as it allows the nucleotide distributions to be conditional on a subset of indexes in the preceding size *w *window (see Figure 1).

To train an IMM on a set of sequences, consider each *w*+1 sized window in the sequences and let the distribution of nucleotides at position *i *in the windows define random variable *X_i_*. Training creates a probabilistic decision tree using information gain as the splitting criteria where each node specifies certain nucleotides at a subset of the window positions and defines a probability distribution for the final nucleotide in the windows. To construct this tree, first, compute the mutual information *I*(*X_i_*; *X_w+1_*) between the final position in the window and positions *i *∈ 1..*w*. Define the initial split in the tree at the position with the greatest mutual information. Create branches to new nodes for all four nucleotides at this position. Next, perform a similar procedure for each branched node considering only windows containing the specific nucleotide at the position chosen. For these windows, compute the conditional mutual information of the remaining positions and choose the most informative position for the next split. Repeat this procedure to fill in the full decision tree, stopping early on paths where data becomes too sparse. At some point walking down each path, additional nucleotide positions may fail to be informative. We recognize this by computing a chi-square test between each node's distribution and its parent node's distribution. If the distributions are sufficiently similar, we stop branching and interpolate between the node and its parent's distributions, weighting each one based on the chi-square test result and the number of training windows mapped to the node.

To compute the likelihood that a novel sequence was generated by this IMM, consider each window of size *w *+ 1 in the sequence as in Equation 1. For each window, follow a path through the decision tree to a leaf node according to the nucleotides at the positions defined by the nodes and branches. Score the next nucleotide in the novel sequence using the leaf node's interpolated probability distribution. More details of the training of IMMs and sequence likelihood computations can be found in the Glimmer descriptions [46,47].

### *K***-means clustering framework**

The *k*-means algorithm is a widely used, simple and effective method for clustering data points. We review that algorithm before introducing our own approach to clustering sequences. Points are modeled as having come from *k *sources, each represented by a cluster mean. The algorithm begins by initializing these cluster means, e.g. by randomly choosing *k *data points. Next, one repeats the following steps. First, compute the distance between all points and the *k *cluster means. Second, assign each point to its nearest cluster.

Finally, recompute the cluster means using the current assignment of points to clusters. After a number of iterations, one arrives at a stable partitioning of data points that approximates the minimum sum of squared distances between data points and their assigned cluster means.

An alternative formulation of the algorithm leads more directly to our approach. The *k*-means algorithm has also been referred to as Classification Expectation-Maximization (CEM) to optimize the Classification Maximum Likelihood (CML) criterion for data points generated from *k *Gaussian distributions with equal variance and zero covariance mixed in equal proportions [48]. For data points *x*_1..._*x_n _*sampled from clusters *C*_1..._*C_K _*and Gaussian density *f *parameterized by mean vectors *u*_1..._*u_K _*, CML is defined as

(2) $$CM\;L(C,u)=\sum_{k=1}^{K}\sum_{x_{i}\in C_{k}}log(f(x_{i};u_{k}))$$

That is, CML approximates the log likelihood that the cluster models generated the data points, but with each data point assigned a hard classification to a single cluster. CML can be further generalized to the case where data points are sampled from the clusters according to a multinomial distribution parameterized by *p*_1..._*p_K _*. Here CEM assigns each data point *x_i _*to the cluster *C_k _*that provides the greatest posterior probability *log*(*p_k _f*(*x_i_*; *u_k_*)), and CML is defined as

(3) $$CM\;L(C,p,u)=\sum_{k=1}^{K}\sum_{x_{i}\in C_{k}}log(p_{k}f(x_{i};u_{k}))$$

Using CEM, the CML criterion converges to a local maximum [48].

#### SCIMM

**Figure 2:**
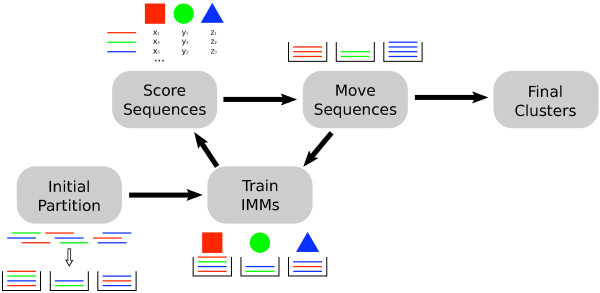
**SCIMM pipeline**. To initialize the IMMs, we initially partition a subset of the sequences into *k *clusters with a previously published method such as CompostBin [39] or LikelyBin [40]. We train an IMM on each cluster, and then compute the likelihood that each sequence was generated by each IMM for all sequences and all IMMs. Next, we reassign each sequence to the cluster corresponding to the IMM which generated it with greatest likelihood. If > 0.1% of the sequences changed clusters, we repeat the process. Otherwise we consider the clusters to be stable and halt.

SCIMM uses the same general algorithm as CEM, where the data points are DNA sequences and the cluster models are IMMs. Here the goal is to find the IMMs and multinomial probabilities that maximize the CML criterion, which approximates the log likelihood that the mixture of cluster models generated the sequences. The algorithm begins by initializing *k *IMMs (discussed in detail below). Then the following steps are repeated until convergence. First, for all sequences *s *and all IMMs *m*, compute the log likelihood that *s *was generated by *m*. Second, assign each sequence to the cluster corresponding to the IMM *m *that maximizes the posterior probability *log*(*p_m_*) + *log*(*P *(*s*|*m*)). Finally, re-train the IMMs on the sequences currently assigned to their corresponding clusters. This loop is depicted in Figure 2. Over the course of the iterations, the IMMs converge to a set that should represent the phylogenetic sources.

Because the Maximization step is not straightforward maximum likelihood estimation (instead using IMM training's highly effective heuristics to choose a model order and interpolate between models), we lose the theoretical guarantee of CML convergence [48]. In practice, we did not find this to be a problem as the algorithm converged in all experiments. However, SCIMM halts when fewer than 0.1% of the sequences change clusters in order to reduce computation time because the last few stages of this procedure tend to shuffle a small number of sequences with a negligible effect on clustering accuracy.

### Initial partitioning

SCIMM inherits the simplicity and effectiveness of the *k*-means algorithm, but also its sensitivity to initial conditions. We found that the likelihood landscape is riddled with local maxima from which the optimization cannot escape. Initially partitioning the sequences by very simple clustering algorithms yielded insufficient results.

To improve performance, we tried using previous methods for unsupervised clustering of metagenomic sequences to initialize the IMMs. We focused on two particularly successful approaches, LikelyBin and CompostBin. LikelyBin models sequences using *k *fixed 2^nd^-4^th ^order Markov models learned by counting oligonucleotides [40]. Because LikelyBin uses simpler models with far fewer parameters than IMMs, a Markov chain Monte Carlo algorithm is used to search the parameter space for the parameters that maximize the likelihood of generating the sequences. LikelyBin is publicly available at http://ecotheory.biology.gatech.edu/downloads/likelybin. The second approach, CompostBin [39], works as follows. For each sequence, count oligonucleotide frequencies and project the frequency vectors into three dimensions using principal component analysis. Next, create a graph where each sequence is represented by a vertex and edges are placed between a sequence and its six nearest neighbors. Finally, split the sequences into two partitions by finding a minimum normalized cut in this graph across which few edges exist [49]. This process is repeated until the desired number of clusters is reached. Though CompostBin is publicly available from http://bobcat.genomecenter.ucdavis.edu/souravc/compostbin, we re-implemented the main unsupervised ideas of the algorithm to better fit in our pipeline and refer to our version as CBCBCompostBin. One notable adjustment to the method was to make the number of nearest neighbors with which to build the graph a function of the number of sequences, because fewer sequences required a less connected graph for good performance. Choosing the number of nearest neighbors is a difficult subproblem of the normalized cut clustering method upon which CompostBin is based [49]. We found that the function $f(n)=2+\frac{1}{2}\left\lfloor \ln(n)\right\rfloor$, where *f *returns the number of nearest neighbors and *n *is the number of input sequences, worked well in practice, but did not address this problem in depth because experimental results demonstrated that SCIMM's accuracy did not depend significantly on the parameter choice.

To initialize the IMMs for SCIMM, we can run either LikelyBin or CBCBCompostBin on a random subset of the sequences with a user-specified number of clusters *k *and train an IMM on every cluster returned. We used a random subset because both algorithms can be slow for large data sets, and 2-3 Mb of sequence was sufficient to train the IMMs to begin the iterative clustering procedure. Because the two programs approach sequence clustering differently, they tend to succeed on different datasets -- e.g. for mixtures of 10 genomes, the standard deviation of the difference between LikelyBin's and CBCBCompostBin's precision (defined below) is 8.3% and recall is 6.5%. Therefore, we initially partition the sequences with both LikelyBin and CBCBCompostBin and perform one iteration of Scimm on each. For each partitioning, we compute the CML criterion and continue iterating on only the partitioning with the greater value.

### Supervised initial partitioning

As we will show, unsupervised clustering methods are very effective on low complexity datasets, but less accurate on metagenomic samples with many (e.g. >20) microbial strains. With more strains, the genome signatures may blend together and become difficult to properly discern. Alternatively, classification methods like Phymm are immune to the complexity of the dataset because each sequence is classified independently of the others [38]. Sequence classifications can be interpreted as implying a clustering, for instance by forming clusters from all sequences classified to the same genus. Therefore, a classification method can also be used to obtain an initial partitioning for SCIMM.

We considered a hybrid of supervised and unsupervised learning referred to as PHYSCIMM where we obtained an initial partitioning of the sequences with Phymm. First, we randomly chose a subset of sequences (again due to computation time concerns and the sufficiency of a subset), classified the sequences, and clustered at a certain taxonomic level (family in our tests). Due to misclassification noise, Phymm will usually return too many clusters. To filter out clusters of misclassified sequences, we found only keeping clusters containing $>\frac{20}{k}\%$ of the sequenced bases where *k *is the number of genomes in the mixture (e.g. > 1% for 20 genomes) to be a useful heuristic. Note that Phymm is not limited to returning *k *clusters, and the number of clusters returned depends on the strictness of filtering, which the user would need to specify in a novel environment. After filtering clusters, we moved all unclustered sequences to an additional cluster, otherwise SCIMM tended to incorrectly force these sequences into the generally high quality clusters from Phymm classifications. Finally, we iterated IMM clustering as in the standard SCIMM algorithm. SCIMM and PHYSCIMM are available open source from http://www.cbcb.umd.edu/software/scimm under the Perl Artistic License http://www.perl.com/pub/a/language/misc/Artistic.html.

## Results and Discussion

### Simulated reads

To assess the performance of SCIMM and PHYSCIMM, we simulated sequencing reads from mixtures of 1028 sequenced genomes in GenBank [19] as of 2009 and clustered the reads with each method. The degree to which the diversity of a random mixture of these genomes is representative of a real metagenomic environment has not been explored in depth. We make two points in support of this experimental setup. First, because certain model and disease-related organisms are of particular interest to researchers, GenBank contains many clusters of extremely closely-related genomes that make clustering difficult and may be representative of a heterogeneous species population from a real metagenomic environment; for example, 29 *Escherichia coli*, 16 *Salmonella enterica*, and 15 *Staphylococcus aureus *genomes were included. Second, while the expected clustering accuracy of any single method on a novel metagenomic environment may not exactly match the statistics reported in our tests, the simulations still serve to rank SCIMM and previous unsupervised approaches based on clustering accuracy.

For each test, we randomly chose *k *genomes and *k *corresponding uniformly distributed random numbers in the interval (0,1). We simulated 30000 reads of length 800 base pairs (bp) so that the percentage of reads from each genome in the sample was proportional to that genome's random number. We clustered the reads with SCIMM, LikelyBin, and CBCBCompostBin. LikelyBin runs used 2 MCMC start points and a 3^rd ^order Markov model. CBCBCompostBin runs used 5-mers.

Clustering accuracy can be quantified using a variety of measures [50]. Sequences from the same genome should be placed in the same cluster, which is measured by *recall*. Let *c_ij _*be the number of sequenced nucleotides from genome *j *placed in cluster *i*. Then the recall for genome *j *is computed as

(4) $$recall(j)\;=\frac{\max_{i}c_{ij}}{\sum_{i}c_{ij}}$$

Sequences placed in a cluster should belong to the same genome. This is measured as *precision *and computed for cluster *i *as

(5) $$precision(i)=\frac{\max_{j}c_{ij}}{\sum_{j}c_{ij}}$$

In order to obtain global performance statistics, precision and recall were combined across clusters and genomes by weighting each term by the number of sequenced nucleotides from the cluster or genome. We also measured accuracy using the adjusted Rand index. The Rand index is the proportion of pairs of data points that are correctly placed together or apart, and the adjusted Rand index modifies this statistic based on the sizes of the clusters [51].

**Figure 3:**
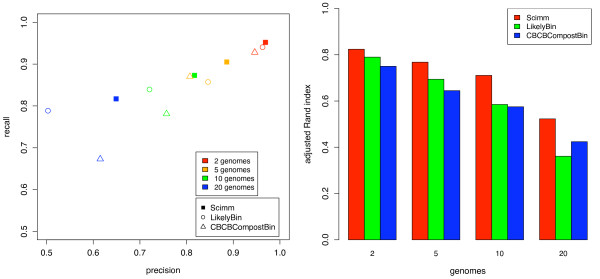
**Unsupervised accuracy**. Cluster accuracy statistics for unsupervised methods on simulated 800 bp reads from random mixtures of genomes in random proportions. SCIMM outperforms the other methods on all tests by all measures.

We tested the unsupervised methods with mixtures of 2, 5, 10, and 20 genomes, performing 40 trials of each, which resulted in standard deviations of ~1.0% for precision and recall and ~1.5% for adjusted Rand index. SCIMM achieved superior performance over the other methods by all measures, as shown in Figure 3. In addition to having a greater average adjusted Rand index, SCIMM had the highest adjusted Rand index for 93% of the trials with ten genomes and 90% of the trials with twenty genomes. All methods were able to effectively partition sequences from two genomes. As we increased the number of genomes, performance degraded, but recall and precision >80% on average can be expected for mixtures of up to ten genomes.

We also examined the effect of sequence length on SCIMM's performance (see Table 1) by sampling mixtures of five and ten genomes and varying the length of the simulated reads while holding the total number of sequenced bp constant; e.g. doubling the number of reads while halving the sequence length. Second generation sequencing technology from Roche/454 produces 400 bp reads, which should be sufficient for clustering sequences from low complexity environments with five or fewer strains as precision and recall are >85%. Accuracy continues to improve with 1600 bp fragments in both the five and ten genome tests, suggesting that longer read lengths or assembly of reads into contigs should be beneficial to metagenomic analysis.

**Table 1:** Varying read length

Length	Genomes	Recall	Precision	Adj Rand
400	5	0.858	0.863	0.689

800	5	0.905	0.886	0.768

1600	5	0.936	0.905	0.817

400	10	0.801	0.756	0.606

800	10	0.869	0.810	0.696

1600	10	0.911	0.821	0.738

In tests with mixtures of five and ten random genomes in random proportions, increasing read length leads to greater accuracy. Even with 400 bp reads, the clusters are accurate enough to be useful for some applications.

All computational methods working with DNA sequencing reads must account for sequencing errors. We expect IMMs to be robust to such errors. A mis-sequenced nucleotide may affect the probabilities of up to *w*+1 nucleotides for window size *w*. However, the IMM will learn which positions in the window are informative for the distribution of the next nucleotide, and errors at uninformative nucleotides will have a negligible effect. Furthermore, even at what are considered high error rates, sequencing errors are rare enough to not overwhelm the genome signatures found in the sequences. To measure the effect of sequencing errors, we sampled mixtures of ten genomes and mis-called nucleotides in the reads at rates of 0.5%, 1.0%, and 2.0%. Table 2 summarizes 40 iterations of this test, such that the standard deviations of precision and recall are ~1.0% and adjusted Rand index is ~1.5%. Though clustering accuracy decreases slightly with errors, increasing the error rate further has a negligible effect, and altogether SCIMM appears to be fairly robust to sequencing errors.

**Table 2:** Varying error rate

Error rate	Recall	Precision	Adj Rand
0.000	0.869	0.810	0.696

0.005	0.852	0.794	0.672

0.010	0.856	0.780	0.665

0.020	0.861	0.782	0.668

In tests with mixtures of ten random genomes in random proportions, sequencing errors lead to decreased accuracy. However, the rate at which accuracy decreases as errors increases is slow so that SCIMM is fairly robust to error rates of 2.0%.

**Figure 4:**
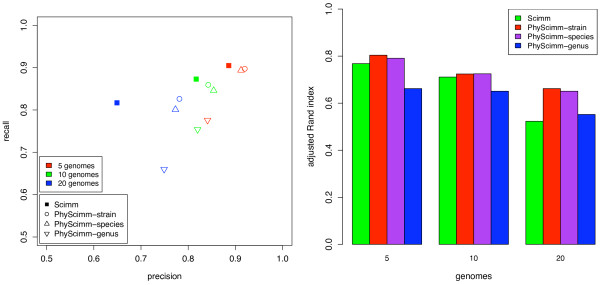
**PHYSCIMM accuracy**. Cluster accuracy statistics for PHYSCIMM on simulated 800 bp reads from random mixtures of genomes in random proportions. PHYSCIMM-strain indicates that IMMs were held out if they matched the strain of a genome in the sample; similarly with PHYSCIMM-species and PHYSCIMM-genus. PHYSCIMM outperforms SCIMM in terms of precision unless IMMs are held out at the genus level and the mixture contains ten or fewer genomes.

Unsupervised clustering performance degrades as the number of genomes reaches twenty or more, but classification methods like Phymm are largely unaffected by the number of genomes. We re-ran the experiment above using PHYSCIMM for mixtures of 5, 10, and 20 genomes. In order to thoroughly evaluate the performance of this supervised initial partitioning of the sequences, we performed separate tests of PHYSCIMM where Phymm's trained IMMs were held out if they were based on the same strain, species, and genus classification as the genomes from which the reads were simulated. For example, if we held out IMMs at the genus level, no IMMs were used from microbial strains matching the genus of any of the genomes from which the reads were simulated. When IMMs from the same genus as those in the sample can be expected, PHYSCIMM produces accurate clusters (see Figure 4). But performance suffers when IMMs are unavailable from the same genus, and unsupervised clustering appears to be more useful in this case at ten and fewer genomes. With few genomes, accuracy is comparable to unsupervised SCIMM, but the value of PHYSCIMM is readily apparent on the twenty genome mixture representing a more diverse metagenomic sample where performance is better than SCIMM even when IMMs are held out at the genus level.

### FAMeS

Experiments clustering single datasets can teach us about specific strengths and weaknesses of the methods and how they can be applied most effectively. To use more realistic data, we clustered the Arachne-assembled contigs from the FAMeS simulated metagenomic datasets of low (simLC) and medium (simMC) complexity [13]. These were created by mixing real reads from the original sequencing projects of 113 organisms. The contigs are dominated by a few species, but have a long tail of very low abundance species. We clustered with SCIMM using *k *= 2-6 clusters and with PHYSCIMM initializing clusters from genus level classifications assigned to >1% of the total bp. Because Phymm has trained IMMs for these publicly available genomes, we held out IMMs similar to organisms in the mixture at the strain and species levels. In a noisy dataset with many organisms like this one, sequences from different strains of the same species are effectively indistinguishable. Thus, we computed accuracy at the species level for the tests that follow.

**Figure 5:**
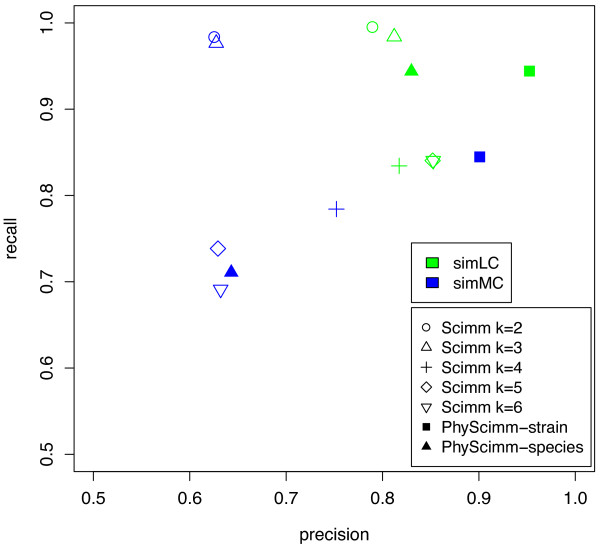
**FAMeS accuracy**. Cluster accuracy statistics for FAMeS Arachne-assembled contigs. We ran SCIMM at a range of values for *k*, the number of clusters, resulting in consistently high accuracy on the low complexity simLC dataset and variable accuracy on the medium complexity simMC for reasons discussed in the text. We ran PHYSCIMM, ignoring IMMs matching genomes in the mix at the strain and species levels (PHYSCIMM-strain and PHYSCIMM-species above). When some IMMs from the same species can be expected, accuracy is far greater, demonstrating that clustering with supervised help relies on models for similar genomes.

The simLC dataset contains 2362 contigs of mean size 3417 bp from 47 different microbial strains, but is dominated by 1283 contigs from *Rhodopseudomonas palustris HaA2 *that make up 73.8% of the nucleotides and 617 contigs from *Bradyrhizobium sp. BTAi1 *that make up 16.3%. The clustering accuracy statistics depend significantly on the arrangement of contigs from these two strains. Because *Rhodopseudomonas palustris HaA2 *and *Bradyrhizobium sp. BTAi1 *are both from the family *Bradyrhizobiaceae *and have similar high GC content (66.0% and 64.9%), separating each strain into its own cluster is difficult. Figure 5 displays the results for both methods. When clustering with SCIMM at *k *= 2 and 3, nearly all contigs from the same strain were kept together leading to 99% recall, but each cluster contained a mix of species giving 80% precision. At larger values of *k*, some reads from the *Bradyrhizobiaceae *strains break off into other clusters, reducing the recall, though at the benefit of increased precision. When holding out IMMs from the same strains, PHYSCIMM achieved very high accuracy (95% precision and 94% recall), as *Rhodopseudomonas palustris HaA2 *and *Bradyrhizobium sp. BTAi1 *were mostly separated from each other because Phymm had a trained IMM for each species. When IMMs from genomes matching species in simLC were removed, PHYSCIMM's precision dropped to 83%. If the initial clusters are formed from Phymm classifications at the family level, the *Bradyrhizobiaceae *strains cannot be separated and precision also drops to 83%.

SimMC has 7307 contigs of mean size 2332 bp from 51 microbial strains. These contigs are distributed among the strains slightly more uniformly, but still only six species account for 99.0% of the nucleotides. These six include two strains each from the species *Rhodopseudomonas palustris *and *Xylella fastidiosa*. *Bradyrhizobium sp. BTAi1 *also appears and presents a challenge similar to that described above for simLC. For *k *= 2 and 3, SCIMM formed strong clusters for the the *Xylella fastidiosa *strains and the *Bradyrhizobiaceae *strains, leading to very high recall. As we increased *k*, these strains were split among the clusters, significantly decreasing the recall (see Figure 5). From this experiment and the last one, we see that clustering performance is best when *k *is set to the number of dominant phylogenetic sources. Increased values of *k *risk splitting a dominant species into multiple clusters rather than effectively clustering a far less abundant species. Precision did not increase with more clusters because when the *Bradyrhizobiaceae *strains split into multiple clusters, each one contained a mixture of both species. When IMMs from the same species were available, PHYSCIMM produced much better clusters with 90% precision and 85% recall. But, accuracy dropped precipitously when IMMs were held out at the species level. Thus, we see again that PHYSCIMM clusters more accurately if very related genomes are available for training, but pure unsupervised clustering is preferable for a metagenome containing organisms whose taxa are unsequenced.

Instead of computing accuracy at the species level, we could consider a higher level in the hierarchy of taxonomic classification, such as the family level. By doing so, we reward the clustering algorithm for clustering together two sequences that originated from different strains in the same family, such as *Bradyrhizobium sp. BTAi1 *and the *Rhodopseudomonas palustris *strains. Family level precision is >97% in all tests, meaning that generally when SCIMM is merging two separate species into a cluster, they are phylogenetically related.

### In vitro-simulated metagenome

To further explore the effectiveness of SCIMM and PHYSCIMM on more realistic data, we clustered sequencing reads from an *in vitro*-simulated microbial community [52]. Here, ten microbes were mixed into a simulated metagenome and sequenced using a number of different protocols and sequencing techniques. These ten were chosen to cover a wide range of microbial diversity, but also to include closely related species, specifically two *Lactobacillus *strains and two *Lactococcus *strains. The resulting reads were then assigned to their source genome via BLAST [18] alignments to a database of the ten microbes' genomes. After combining classified reads from all non-454 datasets, we obtained 24410 mated reads and 3285 singleton reads.

We clustered the reads with SCIMM into 8 clusters for the 8 species in the data and computed 87% recall and 88% precision at the species level. As expected, the *Lactobacillus *and *Lactococcus *strains each clustered together well. High quality clusters formed around the medium abundance strains *Shewanella amazonensis SB2B *and *Myxococcus xanthus DK 1622*, but SCIMM split reads from the most abundant strain *Acidothermus cellulolyticus 11B *into mainly two clusters. Meanwhile, low abundance strains *Pediococcus pentosaceus ATCC 25745 *and *Halobacterium sp. NRC-1 *lacked the data to form their own pure clusters and co-clustered with the *Lactococcus *strains and *Acidothermus cellulolyticus 11B *respectively. Knowing that SCIMM can struggle with low abundance species and seeing that 2 of the 8 clusters were effectively unused and contained far fewer reads than the rest, we reduced the number of clusters to 6. Doing so brought the two *Acidothermus cellulolyticus 11B *clusters together and increased the recall to 94%.

Clustering the reads with PHYSCIMM led to further insight. The level at which IMMs were held out did not have a significant impact on clustering accuracy for this dataset, so we discuss the results from holding out IMMs from the same genus as the strains in the simulated metagenome. We initially clustered sequences using family classifications that were assigned to >3% of the sequences. Performance was considerably worse than unsupervised SCIMM with a 79% recall and 83% precision on the 7 clusters.

PHYSCIMM struggled with *Acidothermus cellulolyticus 11B *because there are no other trained IMMs in its family *Acidothermaceae*. Instead, Phymm assigned its reads to the families *Mycobacteriaceae*, *Microbacteriaceae*, and *Propionibacteriaceae*. Each of these families belong to the order *Actinomycetales*, and so PHYSCIMM performed far better when initialized using order classifications (6 clusters with 88% recall and 92% precision). Interestingly, Phymm misclassifies many reads from the *Lactococcus *strains, the *Lactobacillus *strains, and *Shewanella amazonensis SB2B *to the order *Enterobacteriales*, but iterative IMM clustering is able to effectively separate these species despite a poor initialization.

## Conclusions

Determining the relationships between sequences is a crucial step in metagenomics analysis. In this paper, we introduce SCIMM, an unsupervised sequence clustering method based on interpolated Markov models (IMMs). Our experiments show that SCIMM clusters sequences more accurately than previous unsupervised algorithms.

By demonstrating the ability of IMMs to successfully cluster sequences, we add to the growing evidence of the effectiveness of IMMs for modeling DNA sequences [38,53]. Markov chain models have proven to be useful sequence modeling tools for many bioinformatics applications [45]. The increased modeling sophistication and ability to handle varying amounts of training data make IMMs preferable for many of these applications.

We compared two variations of clustering with IMMs. SCIMM is purely unsupervised and makes use of the previously published methods LikelyBin and CompostBin to initially partition the sequences. PHYSCIMM partitions the sequences using supervised Phymm classifications before the unsupervised iterative IMM clustering stage. Supervised learning proved to be a valuable addition to the pipeline when genomes were available to train on from the same genus as the microbes in the mixture. Pure unsupervised learning is preferable when the available genomes to train on are not representative of those from which the sequencing reads originated. Because the classification accuracy of Phymm is independent of the complexity of the mixture, supervised learning also improves clustering of complex mixtures of twenty or more microbes. Developing more sophisticated combinations of classification and clustering methods may prove to be a fruitful line of research.

We believe SCIMM and PHYSCIMM will be valuable tools for researchers seeking to determine the relationships between sequencing reads from a metagenomics project. For environments with ten or fewer species, unsupervised clustering with SCIMM finds accurate clusters. However, the number of clusters *k *must be chosen carefully by the user. Specific knowledge about the environment, especially regarding the number of dominant microbes and their relationships to each other, can inform the choice of *k *and impact the utility of a clustering of the environment's sequences. Nevertheless, tests on the FAMeS dataset showed that various values of *k *can produce useful clusters. Lesser values of *k *tend to provide greater recall with lower precision. Greater values of *k *may decrease recall by dividing a particularly dominant species into more than one cluster but will usually improve precision.

When the microbes in an environment are thoroughly represented in public databases, PHYSCIMM finds even more accurate clusters. PHYSCIMM is also more effective for mixtures of twenty or more strains. The user does not need to choose the number of clusters with PHYSCIMM, but must choose the classification level and minimum support to initialize a cluster. The simulated read experiments offer guidelines for how to set these parameters effectively. Tests with the FAMeS and in vitro-simulated metagenome datasets demonstrated that incorporating knowledge about the dominant organisms in the environment can have a significant positive impact on the clusters.

Metagenomics projects are increasingly turning to less expensive and higher throughput second generation sequencing technologies such as those from Roche/454 and Illumina. Clustering of 400 bp read lengths is still reasonably effective for mixtures of five or fewer strains. Shorter reads, such as the 100-125 bp lengths currently available from Illumina, cannot be accurately clustered by our methods for environments with realistic complexity. However, if these reads can be assembled into larger contigs, then effective clustering of the contigs is possible.

A number of avenues appear worthwhile for further research. A principled method for setting parameters that affect the number of clusters would certainly aid researchers using the method. Preliminary sequencing of 16 S rRNA or other marker genes followed by clustering may effectively achieve this goal [17,54]. The *k*-means iterative clustering framework used by SCIMM works well with a good initial partitioning of the sequences, but other optimization methods might prove more robust to the initial conditions and less prone to getting stuck in local maxima. Because SCIMM nearly always improves on the clustering results of LikelyBin and CBCBCompostBin, there is reason to believe that it would also improve on initial clusters from more accurate future methods. We excluded an interesting feature from the original CompostBin in our experiments whereby reads containing informative marker genes were identified and classified using AMPHORA [17] and the classifications were used to add supplemental edges to the nearest neighbor graph. A similar semi-supervised scheme could be implemented in SCIMM as well. Finally, assembly and clustering are both important steps in metagenomics pipelines, and further exploration of the relationship between the two has the potential to improve both tasks.

## Authors' contributions

DRK conceived, implemented, and tested the method. DRK and SLS wrote the manuscript.
